# An attentional and working memory theory of hallucination vulnerability in frontotemporal dementia

**DOI:** 10.1093/braincomms/fcae123

**Published:** 2024-04-20

**Authors:** Emma M Devenney, Nga Yan Tse, Claire O’Callaghan, Fiona Kumfor, Rebekah M Ahmed, Jashelle Caga, Jessica L Hazelton, James Carrick, Glenda M Halliday, Olivier Piguet, Matthew C Kiernan, John R Hodges

**Affiliations:** Brain & Mind Centre, The University of Sydney, Sydney 2050, Australia; Neurology Department, Western Sydney Local Health District, Sydney 2145, Australia; Brain & Mind Centre, The University of Sydney, Sydney 2050, Australia; Systems Lab, Department of Psychiatry, The University of Melbourne, Parkville 3052, Australia; Brain & Mind Centre, The University of Sydney, Sydney 2050, Australia; School of Medical Sciences, Faculty of Medicine and Health, The University of Sydney, Sydney 2050, Australia; Brain & Mind Centre, The University of Sydney, Sydney 2050, Australia; School of Psychology, The University of Sydney, Sydney 2050, Australia; Brain & Mind Centre, The University of Sydney, Sydney 2050, Australia; Memory and Cognition Clinic, Institute of Clinical Neurosciences, Royal Prince Alfred Hospital, Sydney 2050, Australia; Brain & Mind Centre, The University of Sydney, Sydney 2050, Australia; School of Psychology, The University of Sydney, Sydney 2050, Australia; Memory and Cognition Clinic, Institute of Clinical Neurosciences, Royal Prince Alfred Hospital, Sydney 2050, Australia; Cognitive Neuroscience Center (CNC), Universidad de San Andrés, Buenos Aires B1644BID, Argentina; Latin American Brain Health Institute (Brain Lat), Universidad Adolfo Ibáñez, Santiago 7941169, Chile; Brain & Mind Centre, The University of Sydney, Sydney 2050, Australia; Brain & Mind Centre, The University of Sydney, Sydney 2050, Australia; School of Medical Sciences, Faculty of Medicine and Health, The University of Sydney, Sydney 2050, Australia; Brain & Mind Centre, The University of Sydney, Sydney 2050, Australia; School of Psychology, The University of Sydney, Sydney 2050, Australia; Neuroscience Research Australia, Randwick 2031, Australia; Faculty of Medicine and Health, University of New South Wales 2031, Australia; Neurology Department, South Eastern Sydney Local Health District, NSW 2031, Australia; Brain & Mind Centre, The University of Sydney, Sydney 2050, Australia

**Keywords:** frontotemporal dementia, psychosis, hallucination, MRI, *C9orf72*

## Abstract

The rate and prevalence of hallucinations in behavioural variant frontotemporal dementia is well established. The mechanisms for underlying vulnerability however are the least well described in FTD compared with other neuropsychiatric conditions, despite the presence of these features significantly complicating the diagnostic process. As such, this present study aimed to provide a detailed characterization of the neural, cognitive and behavioural profile associated with a predisposition to hallucinatory experiences in behavioural variant frontotemporal dementia. In total, 153 patients with behavioural variant frontotemporal dementia were recruited sequentially for this study. A group of patients with well characterized hallucinations and good-quality volumetric MRI scans (*n* = 23) were genetically and demographically matched to a group without hallucinations (*n* = 23) and a healthy control cohort (*n* = 23). All patients were assessed at their initial visit by means of a detailed clinical interview, a comprehensive battery of neuropsychological tests and MRI. Data were analysed according to three levels: (i) the relationship between neural structures, cognition, behaviour and hallucinations in behavioural variant frontotemporal dementia; (ii) the impact of the *C9orf72* expansion; and (iii) hallucination subtype on expression of hallucinations. Basic and complex attentional (including divided attention and working memory) and visual function measures differed between groups (all *P* < 0.001) with hallucinators demonstrating poorer performance, along with evidence of structural changes centred on the prefrontal cortex, caudate and cerebellum (corrected for False Discovery Rate at *P* < 0.05 with a cluster threshold of 100 contiguous voxels). Attentional processes were also implicated in *C9orf72* carriers with hallucinations with structural changes selectively involving the thalamus. Patients with visual hallucinations in isolation showed a similar pattern with emphasis on cerebellar atrophy. Our findings provided novel insights that attentional and visual function subsystems and related distributed brain structures are implicated in the generation of hallucinations in behavioural variant frontotemporal dementia, that dissociate across *C9orf72*, sporadic behavioural variant frontotemporal dementia and for the visual subtype of hallucinations. This loading on attentional and working memory measures is in line with current mechanistic models of hallucinations that frequently suggest a failure of integration of cognitive and perceptual processes. We therefore propose a novel cognitive and neural model for hallucination predisposition in behavioural variant frontotemporal dementia that aligns with a transdiagnostic model for hallucinations across neurodegeneration and psychiatry.

## Teaser

For the first time in behavioural variant frontotemporal dementia (bvFTD), Devenney *et al*. characterized the neural and cognitive profile associated with hallucination vulnerability. The authors report dysfunctions specific to attentional and visual subsystems, and related distributed brain structures, that dissociate across *C9orf72*, sporadic bvFTD and the visual subtype of hallucinations.

## Graphical Abstract

**Graphical abstract:**
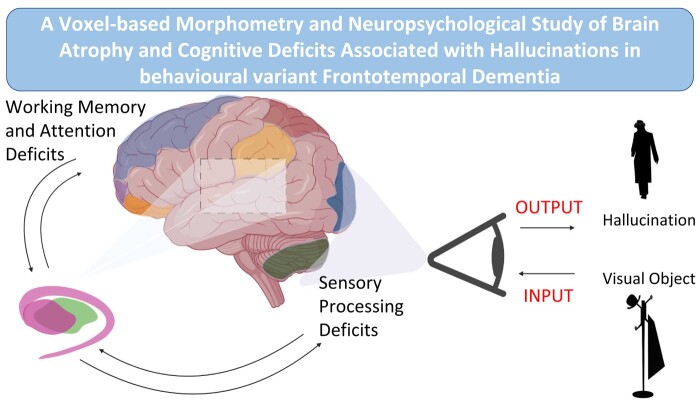

## Introduction

A resurgence of interest in overlapping psychiatric features across neurodegenerative and neuropsychiatric disorders has emerged over the last few years, due to the discovery of the *C9orf72* expansion.^1,2^ It is now well established that psychotic symptoms, including both delusions and hallucinations, are common in behavioural variant frontotemporal dementia (bvFTD) associated with the *C9orf72* expansion but can also occur in sporadic cases, although they are usually less prominent^3-5^ Hallucinations are also present in many other neurodegenerative conditions and perhaps have received the most scientific scrutiny in Parkinson’s Disease^6^ and Lewy Body Dementia.^7^ In bvFTD, these symptoms and the mechanisms for underlying vulnerability are the least well described of all core features, despite placing a burden on carers and significantly complicating the diagnostic process.

Hallucinations in bvFTD are usually negative in nature (e.g. persecutory/negative voices, unpleasant images of deceased people) and encompass perceptual anomalies in the visual, auditory, olfactory and somatic senses.^5^ In bvFTD, these features also extend to more subtle changes of perception across each of the five senses with brain atrophy involving the prefrontal cortex, basal ganglia, thalamus and the cerebellum^8-10^ Relatedly, previous work has shown that patients with bvFTD exhibit schizotypal traits that are considered a risk factor for psychosis, in line with findings of convergent psychotic features in bvFTD and primary psychiatric disorders.^11,12^

While the rate and prevalence of hallucinations in bvFTD have been explored, underlying susceptibility factors have received less attention, compared with Parkinson’s Disease where a model of abnormal attentional processes and related aberrant network connectivity is thought to contribute to hallucinations and has been postulated to extend to Schizophrenia.^13,14^ Separately in bvFTD the thalamus, a key sensory processing and relay station, has been repeatedly shown to be abnormal and associated with psychotic symptoms, in line with documentation from the psychosis literature.^5,15^ Corticothalamic architecture is increasingly implicated as a modulator of attention and conscious perception through widespread connectivity mapped to sensory and motor cortical and subcortical regions^16-18^ Segmentation of the thalamus shows that the *C9orf72* expansion has a predilection for the pulvinar while the mediodorsal nuclei are atrophied across all FTD phenotypes.^19^ In a pathological study of Lewy body disease, the mediodorsal nuclei has been associated with visual hallucinations.^20^ In *C9orf72,* the degree of thalamic atrophy is correlated with cognition and behaviour suggesting a prominent role for the thalamus in *C9orf72* bvFTD.^21^ However, in previous studies of psychosis in bvFTD there was typically a mixture of *C9orf72* carriers and non-carriers and therefore the impact of divergent thalamic involvement in relation to psychosis has not been fully determined.

Similarly, regions involved in multisensory integration including the insula, amygdala, medial temporal regions and the temporoparietal junction, are compromised in schizophrenia. In bvFTD, these regions together with the cerebellum and thalamus may be responsible for alterations in self non-self-differentiation which in turn is associated with the generation of hallucinations and dissociative states^22-26^ Although speculative, these findings suggest that hallucination vulnerability in bvFTD may share some similarities with models from other neurodegenerative and psychiatric conditions.

As such, this present study aimed to explore the neural, cognitive, behavioural and psychosocial factors associated with hallucinations in a well-characterized, and genetically matched sample of patients with bvFTD, to ultimately delineate the cognitive and behavioural profile associated with a predisposition to hallucinatory experiences. We sought to understand and account for the impact of environmental and social factors. Based on the current working models from psychiatry and neurodegeneration, we hypothesized that attentional and working memory deficits may contribute to hallucination generation in bvFTD and that basic sensory impairment may also be involved—together with a pattern of extended brain atrophy that accounts for variability in subtype, presentation and severity.

## Materials and methods

### Participants and group selection

In total, 153 consecutive patients with bvFTD, from the FRONTIER frontotemporal dementia multidisciplinary research clinic specializing in younger-onset dementias at the Brain and Mind Centre, University of Sydney and Neuroscience Research Australia (NeuRA) based in Sydney, Australia, were assessed for inclusion in this study. Standard diagnostic assessment consisted of a clinical and neurological examination, neuropsychological assessment and clinical interview to determine the presence of hallucinations according to DSM-5 criteria. Diagnosis was determined by multidisciplinary consensus in accordance with the current clinical diagnostic criteria for bvFTD and patients were included if they satisfied probable or definite criteria for bvFTD.^27^ Clinical examination was conducted in the presence of a carer where detailed information regarding the previous psychiatric and medical history (i.e. history of illicit substance use, heavy drinking, smoking and psychiatrist consultation, as well as current and previous medications) is obtained. At clinical examination 30 of the 153 (21%) patients presented with symptoms of hallucination (i.e. auditory and/or visual hallucination) at initial clinical consultation, in line with previously reported rates in bvFTD populations. Of these 30 patients, 23 had a brain MRI and were included in the current study. Twenty-three genetically (for *C9orf72*) and demographically matched patients without hallucination and 23 demographically matched healthy controls were included as comparison groups. This approach was favoured to remove the bias of genetic bvFTD in the hallucinations group.

To improve group homogeneity and explore genetic influence and modality-specific variables further sub-analyses were carried out on two additional groups consisting of (i) visual hallucinations alone versus no hallucinations versus controls and (ii) *C9orf72* carriers with hallucinations versus *C9orf72* carriers without hallucinations versus controls.

All controls scored above the cut-off for normal range (>88/100) on the third edition of the Addenbrooke’s Cognitive Examination (ACE-III) ensuring the absence of significant cognitive impairment.^28^ Disease severity was measured by the total score of the Disability Assessment for Dementia (DAD).^29^ In total 13 of the 23 patients presented with a combination of visual and auditory hallucinations, eight patients had visual hallucinations alone, while only two had auditory hallucinations alone. Considering this finding the sub-analyses of hallucination type only focused on those with visual hallucinations alone.

Exclusion criteria for all participants included the presence of other significant neurological or neurodegenerative syndromes. Participants were also excluded from the study if they were prescribed medications known to cause psychotic symptoms or if they were diagnosed with a primary psychotic disorder more than 10 years prior to the onset of their neurodegenerative condition. They were also excluded from the study if they were consumers of any hallucinogenic substances.

### Genotyping

All patients and controls underwent blood sampling to screen for a *C9orf72* repeat expansion. Genomic DNA was extracted from peripheral blood lymphocytes. Proband DNA samples were then screened for the hexanucleotide repeat expansion in the *C9orf72* gene using a repeat primed polymerase chain reaction based on the protocol of Renton *et al*.^1^ Samples were scored as positive if they harboured an allele with more than 30 repeats.

### Ethics statement

The studies involving human participants were reviewed and approved by the South Eastern Sydney Local Health District, the University of New South Wales and the University of Sydney Ethics Committees. The participants/their person responsible provided written informed consent to participate in this study.

### Cognitive measures

All participants completed the ACE-III, a general cognitive screen comprising a total score as well as scores for the attention, memory, fluency, language and visuospatial subdomains.^28^ A total score of less than 88 is indicative of cognitive impairment. Additional cognitive measures of attention (Part A time of the Trail Making Test; TMT^30^); and Maximum Forward Digit Span (immediate attention span),^31^ working memory (Maximum Backward Digit Span),^31^ language (Sydney Language Battery; SYDBAT; comprising of naming, repetition, comprehension and semantics subdomains),^32^ visuospatial skills (Rey Complex Figure copy score),^33^ verbal memory (Rey Auditory learning test delayed recall score; RAVLT);^34^ visual memory (Rey Complex Figure recall score); and executive functions (overall scaled score on Hayling Sentence Completion Test;^35^ number of errors on Part B of TMT; and Part B time—Part A time of TMT) were also administered. Emotion processing was measured using the Face Identify Discrimination Task (FIDT), Face Affect Discrimination Task (FADT), negative and positive emotion total score of Face Affect Selection Task (FAST) from the Face and Emotion Processing Battery.^36,37^

### Measures of behavioural, neuropsychiatric and mood disturbances

To exclude the potential confounding influence of other behavioural and neuropsychiatric impairments, subdomains of the Neuropsychiatric Inventory (NPI; completed by carers of patients)^38^ including agitation, elation, disinhibition, sexual interest changes, apathy, irritability, abnormal stereotypic behaviours, sleep disturbance and appetite changes were included. Composite scores (frequency × severity) were computed for these subdomains and used in subsequent analyses. In addition, all patients completed the self-report Depression Anxiety Stress Scale (DASS)^39^ an inventory of current mood disturbance, to explore potential differences in depressive, anxiety and stress symptomatology between groups.

### Statistical analyses

Data were analysed using SPSS Statistical software, version 24.0. Demographic (i.e. age, education) and cognitive variables were compared across groups (i.e. patients identified as with and without hallucination at initial clinical consultation, and controls) using 1-way analysis of variance (ANOVA) followed by Sidak or Games–Howell *post hoc* tests in case of violation of homogeneity of variances assumption. Categorical variables (i.e. sex and *C9orf72* repeat expansion) were analysed using chi-squared tests. Other clinical (i.e. disease duration, DAD total score) and behavioural (i.e. NPI and DASS subdomain scores) variables specific to patient groups were analysed using independent sample *t*-tests.

When sample sizes were predicted to be small in the sub-analyses exploring neural correlates of hallucination type and hallucination associated with *C9orf72* repeat expansion, the above analyses of continuous variables (i.e. demographic and cognitive variables) were analysed using non-parametric Kruskal–Wallis test, followed by Mann–Whitney *U post hoc* comparisons, while chi-squared test was used for categorical variables (i.e. sex and *C9orf72* repeat expansion). To minimize the risk of Type I errors, statistical significance level was set at a stringent level of *P* < 0.001 for all analyses.

### Imaging

#### Brain imaging acquisition

The majority of the patients (i.e. 19 patients from the hallucination group and 20 from the non-hallucination group) underwent volumetric MRI on a 3T Philips Achieva scanner equipped with a standard 8-channel head coil using the following protocol: matrix 256 × 256, 200 slices, 1 mm^2^ in-plane resolution, slice thickness = 1 mm, echo time = 2.6 ms, repetition time = 5.8 ms, flip angle = 8°. A small subset of patients (i.e. four and three patients from the hallucination and non-hallucination group, respectively) were scanned on a 3T General Electric (GE) scanner with a standard 8-channel head coil using the following protocol: matrix 256 × 256, 200 slices, 1 mm^2^ in-plane resolution, slice thickness = 0.5 mm, echo time = 2.6 ms, repetition time = 5.8 ms, flip angle = 8°. A chi-square was run which confirmed that distribution of the scanner used did not differ between hallucination and non-hallucination groups (*P* = 0.681).

All images were visually inspected by the investigators (E.M.D. and N.Y.T.) where all 23 scans had clear distinction of white and grey matter without the presence of major artefacts (e.g. head movements) and therefore were determined to be of sufficient quality and included in subsequent processing.

#### Voxel-based morphometry analysis

VBM analysis was conducted on the T1-weighted images, using the FMRIB Software Library (FSL) package, version 6.0.0 (http://fsl.fmrib.ox.ac.uk/fsl/fslwiki/FSLVBM). In the first instance, brain extraction was conducted using the BET algorithm in FSL.^40^ Each extracted scan was visually checked to ensure that no brain matter was excluded and no non-brain matter (e.g. dura mater, skull) remained. Brain-extracted images were then segmented into cerebrospinal fluid, grey matter and white matter using the FMRIB Automatic Segmentation Tool (FAST).^41^ Following which, the grey matter partial volumes were non-linearly registered to the Montreal Neurological Institute Standard space (MNI152) using FNIRT with a b-spline representation of the registration warp field.^42^ An equal number of the registered grey matter images from each group was selected and concatenated into a grey matter template specific to this study with nonlinear (nonaffine) registration to ensure equal representation and minimize potential bias. Each voxel of each registered grey matter image was divided by the Jacobian of the warp field to correct for any contraction/enlargement caused by the nonlinear component of the transformation. Smoothing of the segmented and modulated normalized grey matter images was then conducted using a Gaussian kernel of 3 mm.

Whole-brain general linear models (GLMs) were performed to explore neural correlates of hallucination status. First, comparison of cerebral grey matter volume differences was conducted between each patient group and controls (i.e. patients with hallucination versus controls; and patients without hallucination versus controls) using independent *t*-tests. Next, difference in the pattern of whole-brain atrophy was analysed between patient groups (i.e. patients with hallucination versus patients without hallucination). Clusters were extracted using voxel-based method and corrected for False Discovery Rate (FDR) at *P* < 0.05 with a cluster threshold of 100 contiguous voxels to control for family-wise error.

Additional exploratory analyses were conducted to examine the neural correlates of visual hallucination status, as well as hallucination associated with *C9orf72* repeat expansion. Specifically, cerebral grey matter volume differences were compared between 8 patients with visual hallucination, 8 clinically and demographically matched patients without any hallucination and 8 demographically matched healthy controls; as well as between 10 *C9orf72* expansion carriers with hallucination, 9 matched *C9orf72* expansion carriers without hallucination and 10 matched healthy controls. To compensate for the impact of small sample sizes on statistical power, a less conservative significance threshold of *P* < 0.005 (uncorrected for multiple comparisons) was employed for all exploratory analyses. However, a consistent cluster extent threshold of 100 contiguous voxels was applied, which is relatively conservative compared to the uncorrected threshold of 30 contiguous voxels used in past VBM studies in similar clinical populations.^43,44^ In the light of the unique role of the thalamus in hallucination predisposition, thalamic results were overlaid on a high-resolution structural MRI-based atlas of thalamic nuclei^45^ to visualize the patterns of subregional involvement.

## Results

### Demographic characteristics

In total, 19 of 46 (41%) of patients tested positive for the *C9orf72* gene expansion. Across the cohorts, there were no significant group differences between patients with and without hallucination on any of the demographic (i.e. sex, education years, age at scan) or clinical (i.e. disease duration, *C9orf72* repeat expansion status and DAD total score) variables (Table 1), *C9orf72* repeat expansion carriers with and without hallucination (Supplementary Table 1) and patients with and without visual hallucination (Supplementary Table 2; all *P* values > 0.001). All patient groups also did not differ in their psychiatric, substance use or medical history (Supplementary Tables 3–5).

**Table 1:** Demographic characteristics between patients with and without any hallucinations

	Healthy Controls (*n* = 23)	Patients with hallucination (*n* = 23)	Patients without hallucination (*n* = 23)	Test-statistic	*P*
Sex (M/F)	9/14	12/11	15/8	3.136^a^	0.208
Education (years)	13.37(1.66)	12.72(3.32)	11.72(2.31)	2.50^b^	0.092
Age of at scan (years)	63.30(4.61)	61.13(8.40)	59.83(8.77)	1.263^b^	0.291
Disease duration (months)	-	55.36(34.24)	42.09(21.50)	1.540^c^	0.133
*C9orf72* status (Y/N)	-	10/12^d^	9/14	0.184^a^	0.668
DAD	-	50.94(21.26)	61.39(20.20)	−1.689^c^	0.099
Caucasian ethnicity	100%	100%	100%	-	-

Means (Standard Deviation).

ACE, Addenbrooke’s Cognitive Examination; DAD, Disability Assessment for Dementia.

^a^Chi-square value.

^b^Brown–Forsythe’s *F* value.

^c^Independent sample *t*-test.

^d^ *C9orf72* status information was unavailable for one patient in the hallucination group, no other missing values across all other variables.

### Cognitive measures

#### BvFTD cohort

Relative to healthy controls, significantly poorer performance across ACE-III memory and fluency subdomains, language, verbal memory and emotion processing functions were identified in all bvFTD patients, indicative of widespread cognitive impairment in this group regardless of hallucination status (all *P* values < 0.001, Table 2).

**Table 2:** Neuropsychological test performance between patients with and without any hallucinations

Cognitive function	Healthy controls (*n* = 23)	Patients with hallucination (*n* = 23)	Patients without hallucination (*n* = 23)	*F*	*P*	*Post hoc*	*P*
**ACE**							
Total	96.13(2.56)	66.62(12.95)	77.79(15.93)	35.093^a^	<0.001	CRL > HAL, NONHAL	<0.001
Attention	17.35(1.27)	13.42(2.50)	15.89(2.47)	19.002^a^	<0.001	CRL > HAL	<0.001
Memory	25.09(1.31)	15.55(5.94)	19.83(5.23)	23.872^a^	<0.001	CRL > HAL, NONHAL	<0.001
Fluency	12.74(1.25)	5.18(3.46)	6.61(4.18)	35.446^a^	<0.001	CRL > HAL, NONHAL	<0.001
Language	25.35(0.78)	19.87(4.16)	21.42(5.12)	12.261^a^	<0.001	CRL > HAL	<0.001
Visuospatial	15.61(0.72)	12.84(2.35)	14.19(2.28)	11.467^a^	<0.001	CRL > HAL	<0.001
**Attention**							
TMT-A (seconds)	30.32(6.97)	69.74(41.08)	51.32(31.62)	9.132^a^	<0.001	CRL > HAL	<0.001
Forward Digit Span (Max)	7.32(1.45)	5.70(1.06)	6.14(1.68)	6.971^a^	0.002	-	-
**Working memory**							
Backward Digit Span (Max)	5.32(1.16)	3.22(1.00)	4.35(1.57)	14.746	<0.001	CRL > HAL	<0.001
**Language**							
SYDBAT- Naming	27.11(2.08)	18.86(4.26)	21.57(5.46)	20.120^a^	<0.001	CRL > HAL, NONHAL	<0.001
SYDBAT- Repetition	29.89(0.32)	27.33(5.16)	27.48(4.59)	2.509^a^	0.094	-	-
SYDBAT- Comprehension	29.28(1.53)	23.48(4.50)	26.32(4.13)	13.291^a^	<0.001	CRL > HAL	<0.001
SYDBAT- Semantics	28.00(1.68)	21.38(3.99)	25.05(4.85)	15.543^a^	<0.001	CRL > HAL	<0.001
**Visuospatial function**							
RCF Copy (Raw)	33.05(2.63)	21.84(8.80)	25.77(8.23)	12.260^a^	<0.001	CRL > HAL	<0.001
**Executive functions**							
Hayling Total (SS)	6.05(0.97)	2.87(1.60)	4.12(2.26)	15.219^a^	<0.001	CRL > HAL	<0.001
TMT-B (errors)	0.32(0.67)	2.36(1.80)	0.82(1.13)	8.345^a^	0.002	-	-
TMT-B-A (seconds)	40.37(18.21)	220.96(91.95)	126.68(104.01)	26.795^a^	<0.001	CRL > HAL	<0.001
**Visual memory**							
RCF 3-minute delay	17.89(4.66)	7.11(4.79)	11.38(8.91)	13.520^a^	<0.001	CRL > HAL	<0.001
**Verbal memory**							
RAVLT 30-minute delay	11.16(2.46)	3.75(3.00)	5.41(4.18)	24.741^a^	<0.001	CRL > HAL, NONHAL	<0.001
**Emotion processing**							
FAST							
Negative emotions	21.35(3.10)	13.12(4.68)	15.74(4.43)	17.767^a^	<0.001	CRL > HAL, NONHAL	<0.001
Positive emotions	11.59(0.87)	8.53(3.22)	10.68(1.34)	9.672^a^	<0.001	CRL > HAL	0.004
FADT	36.65(1.50)	29.00(5.74)	34.47(4.29)	14.842^a^	<0.001	CRL > HAL	<0.001
FIDT	36.00(3.87)	28.29(5.25)	32.89(5.93)	6.860	0.003	-	-

Significance set at *P* < 0.001.

Means (Standard Deviation). ^a^Brown–Forsythe’s *F* value.

ACE, Addenbrooke’s Cognitive Examination; CRL, Healthy controls; FADT, Face Affect Discrimination Task; FAST, Face Affect Selection Task; FIDT, Face Identity Discrimination Task; HAL, Hallucination group; Hayling, Hayling Sentence Completion Test; NONHAL, Non-hallucination group; RAVLT, Rey Auditory Verbal Learning Test; RCF, Rey Complex Figure; SS, Scaled Score; SYDBAT, Sydney Language Battery; TMT, Trail Making Test.

Of note, significantly lower scores or greater number of errors on measures of attention, working memory, visuospatial skills, visual memory, divided attention and verbal inhibition were selective to the patient group with hallucinations relative to controls (all *P* values < 0.001, Table 2). This indicates the presence of disproportionate modality-specific cognitive impairment in patients presenting with hallucination, centring on basic and complex attention, visual and executive dysfunctions.

#### *C9orf72* Cohort

Overall *C9orf72* carriers with hallucinations presented with a more severe cognitive profile compared to controls than *C9orf72* non-hallucinators compared to controls on measures of attention and divided attention, in addition to memory and language on the ACE-III (all *P* values < 0.001; Supplementary Table 6).

#### Visual hallucination cohort

While comparable cognitive performance was seen between patients with and without visual hallucination, deficit in ACE-III attention subdomain was selectively observed in visual hallucinators (all *P* values < 0.001, Supplementary Table 7).

### Behavioural, neuropsychiatric and mood disturbances

Additional analyses were conducted to ensure that current findings were unlikely to be biased by the potential confounding influence of other neuropsychiatric symptoms common in bvFTD. This confirmed that there were no significant differences in other behavioural or psychological disturbances across all groups (i.e. NPI and DASS subdomain scores; all *P* values > 0.001; Supplementary Tables 8–10; Supplementary Figs 1–3).

### VBM results

Widespread cortical grey matter intensity reduction that is largely consistent with previous neuroimaging literature in bvFTD was found in participants with (Supplementary Fig. 4) and without hallucinations (Supplementary Fig. 5), including those with *C9orf72* repeat expansion (Supplementary Fig. 6 and 7) and visual hallucination (Supplementary Fig. 8 and 9) when compared to controls.^46^ To explore neural correlates of any subtypes of hallucination, visual hallucination and hallucination associated with *C9orf72* repeat expansion, whole-brain grey matter integrity was compared between these groups.

#### BvFTD cohort—patients with VS without any hallucination

**Figure 1:**
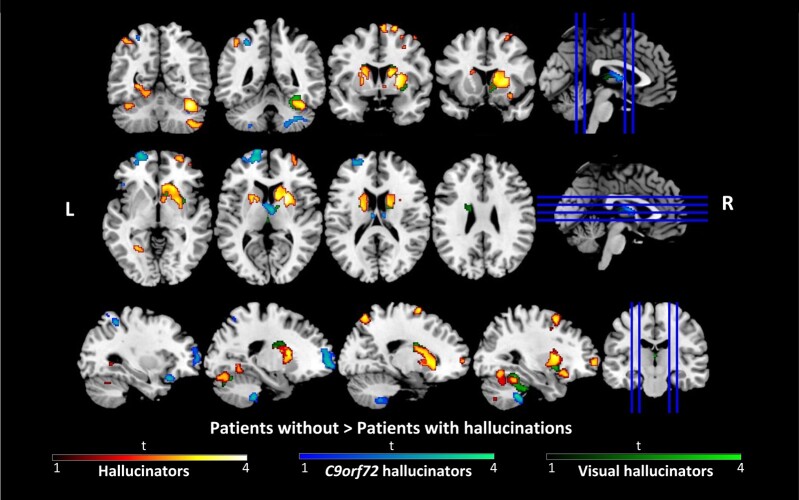
**Voxel-based morphometry analyses showing significant cerebral grey matter intensity differences.** Orange-shaded voxels show regions that were significant between all patients with (*n* = 23) and without any hallucinations (*n* = 23) after correction for False Discovery Rate (FDR) at *P* < 0.05 with a cluster threshold of 100 contiguous voxels. Blue-shaded voxels show regions of greater grey matter intensity reduction in *C9orf72* repeat expansion carriers with hallucination (*n* = 10) compared to carriers without (*n* = 9), and green-shaded voxels indicate regions of greater grey matter intensity reduction in patients with visual hallucinations (*n* = 8) compared to those without any hallucinations (*n* = 8), at the threshold of *P* < 0.005 (uncorrected) with a cluster threshold of 100 contiguous voxels. L, Left; R, Right; *t*, *t* values.

Patients presenting with any hallucination were found to display significant grey matter density reduction in frontal (including frontal orbital cortex, frontal pole and middle and superior frontal gyrus), superior parietal lobule, supramarginal gyrus and occipital (involving occipital fusiform gyrus and lateral occipital cortex) regions, in addition to the striatum (including the caudate and putamen) and the posterior cerebellum (Fig. 1 and Table 3) compared to those without.

**Table 3:** Voxel-based morphometry results of significant cerebral grey matter intensity differences between patients with and without any hallucinations

Group comparison	Cluster size, voxels	MNI coordinates			H	Regions
		X	Y	Z		
**NONHAL > HAL**	1292	40	−72	−52	R	Crus II into Crus I and lobules VI and VIIb
	1281	34	22	−18	R	Frontal Orbital Cortex into caudate, putamen
	485	−26	−56	−2	L	Lingual Gyrus into occipital fusiform gyrus
	341	−14	6	4	L	Putamen into caudate
	212	30	60	0	R	Frontal pole
	203	14	−68	54	R	Lateral Occipital Cortex (superior division) into precuneous cortex
	192	30	8	56	R	Middle frontal gyrus into superior frontal gyrus
	171	12	−2	60	R	Supplementary motor cortex into superior frontal gyrus
	132	−38	−52	50	L	Superior parietal lobule into supramarginal gyrus (posterior division) and angular gyrus
	102	−40	−52	−26	L	Lobule VI into Crus I
**HAL > NONHAL**	NS	-	-	-	-	-

Results were reported at the threshold of *P* < 0.05 (FDR-corrected for family-wise errors) with a cluster threshold of 100 contiguous voxels.

H, Hemisphere; HAL, Hallucination group; MNI, Montreal Neurological Institute; NONHAL, Non-hallucination group.

#### *C9orf72* cohort—carriers with VS without hallucination

**Figure 2:**
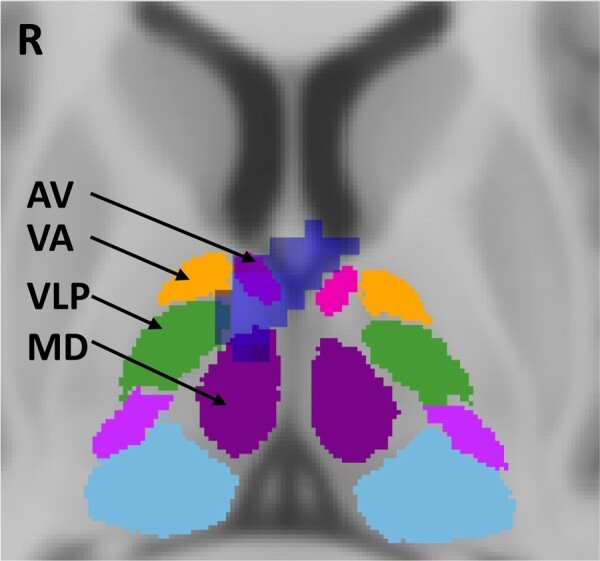
**Thalamic subregional involvement.** An axial view (MNI coordinates: 4, −4, 8) with the right thalamus cluster (in blue) from exploratory analysis of significant grey matter reduction in *C9orf72* repeat expansion carriers compared to carriers without hallucination overlaid on an atlas of the thalamic nuclei.^45^ The cluster boarders on the anteroventral nucleus (AV; pink), the ventral anterior nucleus (VA; orange), the ventral lateral posterior (VLP; dark green) and the mediodorsal nucleus (MD; purple).

Greater reduction in the left frontal pole and frontal orbital cortex, right thalamus, as well as bilateral posterior cerebellum was observed in *C9orf72* carriers with hallucination compared to those without (Fig. 1 and Table 4). Within the thalamus, the cluster was restricted to the anterior and medial thalamus (Fig. 2).

**Table 4:** Exploratory voxel-based morphometry analysis of significant cerebral grey matter intensity difference between C9orf72 repeat expansion carriers with and without hallucination

Group comparison	Cluster size, voxels	MNI coordinates			H	Regions
		X	Y	Z		
**NONHAL > HAL**	507	−20	64	−4	Left	Frontal pole
	230	28	−40	−48	Right	Lobule VIIIb into lobule VIIIa and X and Crus I and II
	172	36	−56	−30	Right	Crus I into lobule VI
	158	6	−8	8	Right	Thalamus
	157	−38	−52	52	Left	Superior parietal lobule into lateral occipital cortex (superior division)
	125	−34	20	−20	Left	Frontal Orbital Cortex
	104	−24	−36	−48	Left	Lobule X into lobule VIIIa

Results reported at the threshold of *P* < 0.005 (uncorrected) with a cluster threshold of 100 contiguous voxels.

H, Hemisphere; HAL, Hallucination group; MNI, Montreal Neurological Institute; NONHAL, Non-hallucination group.

#### Visual hallucinations cohort—patients with VS without visual hallucination

Relative to those without visual hallucination, patients presenting with visual hallucination demonstrated significant reduction in grey matter volume in the bilateral caudate and posterior cerebellum, predominantly in Crus I extending into lobule VI (Fig. 1 and Table 5).

**Table 5:** Exploratory voxel-based morphometry analysis of significant cerebral grey matter intensity difference between patients with and without visual hallucination

Group comparison	Cluster size, voxels	MNI coordinates			H	Regions
		X	Y	Z		
**NONHAL > HAL**	768	16	0	14	Right	Caudate
	542	42	−52	−28	Right	Crus I into lobule VI
	197	−18	−78	−28	Left	Crus I into lobule VI
	139	−18	−2	18	Left	Caudate

Results reported at the threshold of *P* < 0.005 (uncorrected) with a cluster threshold of 100 contiguous voxels.

H, Hemisphere; HAL, Hallucination group; MNI, Montreal Neurological Institute; NONHAL, Non-hallucination group.

## Discussion

This detailed analysis of bvFTD patients with hallucinations proposes a novel cognitive and neural model for hallucination predisposition with selective implications for *C9orf72* expansion carriers and patients experiencing visual hallucinations alone. Across each of the levels of analyses, basic and complex attentional deficits were most consistently associated with the presence of hallucinations. The neuroimaging associations included regions spanning from the prefrontal cortex to subcortical structures with significant involvement of cerebellar structures related to somatosensory and attentional functions. Interestingly, the *C9orf72* analysis implicates the thalamus, as has been previously suggested, however for the first time suggests that thalamic atrophy may be specific or more severe in those with hallucinations, while those with visual hallucinations showed marked cerebellar involvement with neuropsychological evidence for additional deficits in visual processing.

This analysis of hallucinations in bvFTD offers new insights into potential symptom mechanisms that align with a transdiagnostic model for hallucinations across neurodegeneration and psychiatry and proposes that a model that relies on aberrant modulation of top-down processing may be implicated in a predisposition to hallucinations. Although much remains to be explored in this area, this is another step in advancing work that has already been conducted in psychosis and sensory perceptual processing in bvFTD, to consider it in the context of advanced models of perceptual processing, that are already widely regarded across psychiatry and gaining traction in neurodegeneration. This study also highlights the emerging role of subcortical structures in symptom presentation in bvFTD and adds to our understanding of the complex role that the thalamus plays in network coordination, particularly in *C9orf72* bvFTD. That attentional processes are inherent to each of the sub-analysis gives weight to their role in sensory processing in bvFTD.

In bvFTD, there was a clear pattern of cognitive deficits associated with hallucinators with a predominant attentional involvement namely basic and divided attention and working memory, in addition to visual function and inhibitory control. This aligns with evidence from Parkinson’s literature where attention is selectively impaired in those with hallucinations.^13,47^ Attention can be conceptualized as the process involved in the accurate selection of incoming sensory information through a variety of processes including working memory, competitive selection of stimuli and cognitive control, to allow us to function effectively and adapt to changes in our environment.^48,49^ Even in the simple tasks employed in this study, it is obvious that attentional and working memory processes are intricately linked with each having a role in the successful accomplishment of the other^50-52^ This interaction has previously been implicated in hallucination generation and psychosis in general across neuropsychiatric conditions including Lewy Body Dementia.^53,54^ Despite the disparities in cognitive functioning, there were no significant differences in terms of carer-rated behavioural change across all classic bvFTD behaviours including apathy, disinhibition, stereotypy, empathy and changes in appetite in line with previous findings.^5^ This might suggest that these behaviours rely more heavily on higher order cognitive processes, such as social cognition and reward processing, that are not differentially affected across patients with and without hallucinations. An alternative explanation may simply be that these behavioural ratings are less sensitive than objective cognitive markers.

A similar pattern of cognitive change was identified for the genetic and hallucination subtype sub-analyses suggesting that a model that incorporates attentional subsystems is robust across various conditions in bvFTD and indeed measures of attention. This loading on attentional measures is in line with current mechanistic models of hallucination that, although many have been postulated, frequently suggest a failure of integration of top-down cognitive processes and bottom-up perceptual change.^55,56^ Attention as a top-down cognitive process has specifically been implicated in visual hallucination generation.^13,57^ This converges with the prominent association with the thalamus, subcortical structures and the cerebellum as critical regions for sensory and somatosensory processing and with involvement of basic sensory input regions within the occipital lobe.^58,59^ Although it was not directly assessed from a neuropsychological perspective in this study, the findings of atrophy in the supramarginal gyrus in hallucinators support the notion that basic auditory processing may be differentially impaired between hallucinators and non-hallucinators.^60^

**Figure 3:**
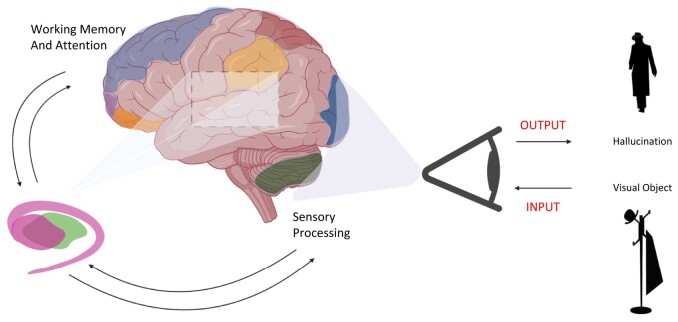
**Schematic representation of top-down and bottom-up processes that influence hallucination generation.** Shaded areas correspond to regions of significant atrophy in hallucinators versus non-hallucinators [middle and superior frontal gyri (light blue), frontal pole (purple), frontal orbital cortex (orange), supramarginal gyrus (yellow), superior parietal lobule (brown), occipital cortex (dark blue), posterior cerebellum (dark green), caudate and putamen (pink), thalamus (green)]. Involvement of subcortical structures as well as cerebellar and occipital cortices suggest impaired sensory processing, while the involvement of frontal and parietal regions indicate higher order attentional deficits. We hypothesize that the contribution of these regions to impaired top-down and bottom-up processes is likely to underly the formation of aberrant percepts relating to sensory evidence e.g. misperception of visual objects leading to complex hallucinations.

Furthermore, in the exploratory sub-analyses of the *C9orf72* cohort, that to our knowledge is the first-time hallucination vulnerability have been compared within the *C9orf72* cohort, we similarly identified greater deficits in attentional processes, encompassing basic and divided attention. Atrophy analysis in this study showed decreased grey matter density in the superior parietal lobule,^2^ which is known to be involved in top-down processes including internal representation of space and attentional orientation and manipulation of spatial information in working memory—suggesting this region along with the thalamus may be critical for hallucination generation in *C9orf72* (Fig. 3).^61,62^ A similar profile emerged for patients with visual hallucinations that included deficits in attention. Overall, these findings converge to provide support for the role of abnormal attentional processing in psychosis manifestation in bvFTD.^8,10^

In bvFTD, the cognitive profile of hallucinators at disease presentation is generally more severe than non-hallucinators (based on the ACE-III global cognition score and that the non-hallucinators did not score poorer than hallucinators on any task) despite a similar disease duration and presumably a similar cognitive reserve based on education levels and similar social circumstances. One relatively simple explanation is that hallucinations signify a different progression of symptoms that is more severe, perhaps based on genetics and or underlying pathology. In this study, we matched the main analysis group for *C9orf72* status to remove the confounds of genetic variability, at least on a single gene level, although other unknown risk genes and epigenetic factors may play a role. Another possibility is that the presence of hallucinations represents an additional cognitive burden in an already struggling system and perhaps the attention subsystems are most sensitive in this regard.

The pattern of atrophy when hallucinators were compared with non-hallucinators showed involvement across critical attentional and working memory regions including the prefrontal cortex, caudate and posterior parietal cortices^63-66^ In Parkinson's disease, the attentional theory of hallucinations focuses on disruption in functional attentional networks.^67^ A similar theory has been proposed in schizophrenia with disruption of the default mode and central executive networks implicated in hallucination generation.^68^ While the nature of network dysfunction related to hallucinations in bvFTD is beyond the scope of this study it seems reasonable to consider, given our knowledge of network dysfunction in bvFTD, particularly affecting the default mode network, that a similar process may be involved to some extent in bvFTD.^69^ In this study, we also see involvement of the cerebellum across each of the analysis steps highlighting a pivotal role for the cerebellum in hallucination generation in keeping with previous findings of cerebellar atrophy related to abnormal sensory processing across the bvFTD-motor neuron disease spectrum.^10^ As expected, in *C9orf72* there is extensive involvement of the cerebellum in hallucinators that is predominantly in cerebellar regions associated with cognitive and affective symptoms rather than motor control.^70^ Finally, as briefly discussed above, the thalamus is a critical hub for sensory processing and in functional attentional networks. Given the role of the thalamus and the marked degree of atrophy in this region in *C9orf72* carriers with hallucinations, it is perhaps not surprising that there was preferential atrophy of the thalamus in *C9orf72* carriers. This would also be in line with mounting literature showing distinct thalamic atrophy in *C9orf72* expansion^19,21,71^ But for the first time, this study raises the possibility that perhaps thalamic atrophy is much more extensive in *C9orf72* carriers with hallucinations, and substantially less in those without (Figs 1 and 2 and Supplementary Fig. 6 and 7). As mentioned in the introduction the thalamus has been repeatedly implicated in psychosis in other neuropsychiatric and neurodegenerative diseases likely through its role in sensory processing and as a key hub in the corticothalamic network. We thus hypothesize that *C9orf72* pathology may selectively target the corticothalamic network, leading to altered connectivity with sensory and motor cortical and subcortical regions that modulate incoming sensory information and top-down cognitive processing and in turn contribute to predisposition to psychotic experience as part of a wider network disintegration. The lack of thalamic atrophy in sporadic cases with hallucinations also suggests that in sporadic bvFTD the mechanism of hallucination may be different and may involve other subcortical structures including the basal ganglia. However, further work remains to be undertaken in advancing our understanding. In bvFTD, there is often significant variability of patterns of grey matter loss and functional connectivity disruptions across brain regions and networks, further compounded by heterogeneity in genetic involvement. It is likely that a number of factors converge to infer a vulnerability to psychotic features that remain to be elucidated.

Several limitations warrant consideration. Firstly, the low rate of psychosis has substantially limited the size of the hallucinatory cohorts, although the prevalence observed in the present study is comparable to that reported in population-based studies (see review^72^). In relation, due to issues with poor reliability of the NPI or other behavioural measures for hallucination severity, the neural correlates of hallucination were derived from between-group comparison analyses. To mitigate the issues associated with this methodology, extensive phenotyping and demographic, clinical and genetic profiling were conducted to ensure the absence of disproportionate differences in any of the potentially confounding variables in the closely matched patient comparison group. Future studies may nevertheless benefit from the use of a subclinical measure such as the Schizotypal Personality Questionnaire (SPQ)^73^ or the Cambridge Anomalous Perceptions Questionnaire (CAPs)^74^ that taps into a wider spectrum of symptoms associated with schizotypy and psychosis proneness, respectively, though neither are specific for FTD. This will allow for not only more detailed psychotic symptom subtyping beyond positive symptoms but also the inclusion of patients with subclinical presentations, facilitating the possibility of a better-powered correlational VBM analysis to verify current findings. All of the participants included in the present study were Caucasian, highlighting the important need for future studies to increase racial diversity in both healthy control and clinical cohorts. The potential impact of the use of two scanners on neuroimaging findings also requires consideration. The chi-square analysis did not reveal significant differences in the distribution of scanners between any of the clinical groups, suggesting potential bias towards a particular scanner was unlikely. Lastly, future studies may consider the use of a multimodal approach incorporating functional neuroimaging to directly examine the contribution of canonical functional networks particularly the default mode and attentional networks as proposed in the current study.

## Conclusion

In conclusion, we have identified a cognitive and neural model associated with hallucinations in bvFTD that may differentiate across genetic and sporadic diseases. These results have implications for patient care and carer support and suggest features that may point clinicians to probe for the presence of hallucinations. This study has also revealed information regarding brain-behaviour relationships and implies that disruption in brain regions outside of the traditional frontal and temporal lobes may have critical roles in symptom generation that can begin early in the disease process—as psychotic symptoms can often precede typical behavioural features in bvFTD. It seems possible that alterations in attentional processes could contribute to disorder within the hierarchical systems involved in sensory processing to generate an ambiguous image, sound, touch or smell (Fig. 3). Our results suggest that in bvFTD the generation of these percepts is complex and likely involves distinct cognitive mechanisms and widespread neural structures.

## Supplementary Material

fcae123_Supplementary_Data

## Data Availability

The data that support the findings of this study are available from the corresponding author on request.
