# Supplementary material for: An attentional and working memory theory of hallucination vulnerability in frontotemporal dementia
Source: Brain Commun. 2024 Apr 20;6(3):fcae123. doi: 10.1093/braincomms/fcae123 (PMC11081077; doi:10.1093/braincomms/fcae123)
Supplement: fcae123_Supplementary_Data [file fcae123_supplementary_data.docx]

**Supplementary Table 1.** Demographic characteristics between *C9orf72* repeat expansion carriers with and without hallucination

|  | **Healthy Controls (*n* = 10)** | ***C9*+ Patients with hallucination**  **(*n* = 10)** | ***C9*+ Patients without hallucination**  **(*n* = 9)** | **Test-Statistic** | ***p*** |
| --- | --- | --- | --- | --- | --- |
| Sex (M/F) | 6/4 | 6/4 | 7/2 | .868^a^ | .648 |
| Education (years) | 13.10(1.97) | 12.80(3.49) | 11.64(2.15) | 2.129^b^ | .345 |
| Age of at scan (years) | 61.70(2.98) | 60.50(7.55) | 57.22(6.65) | 2.688^b^ | .261 |
| Disease duration (months) | - | 54.90(33.03) | 30.75(16.82) | 22.00^c^ | .122 |
| DAD | - | 51.05(24.06) | 65.16(19.82) | 22.500^c^ | .122 |

Means (Standard Deviation). ^a^Chi-square value. ^b^Kruskal Wallis *H* value. ^c^Mann Witney *U* value.

DAD = Disability Assessment for Dementia.

**Supplementary Table 2.** Demographic characteristics between patients with and without visual hallucination

|  | **Healthy Controls**  **(*n* = 8)** | **Patients with visual hallucination**  **(*n* = 8)** | **Patients without any hallucination**  **(*n* = 8)** | **Test-Statistic** | ***p*** |
| --- | --- | --- | --- | --- | --- |
| Sex (M/F) | 2/6 | 4/4 | 3/5 | 1.067^a^ | .587 |
| Education (years) | 12.69(1.49) | 12.19(2.85) | 11.91(1.90) | .590^b^ | .745 |
| Age of at scan (years) | 65.13(5.46) | 59.00(6.85) | 62.38(7.37) | 3.406^b^ | .182 |
| Disease duration (months) | - | 60.86(49.70) | 41.00(11.90) | 27.000^c^ | .955 |
| *C9orf72* status (Y/N) | - | 2/5 | 3/5 | .134^a^ | .714 |
| DAD | - | 60.85(26.44) | 65.11(13.34) | 27.500^c^ | .955 |

Means (Standard Deviation). ^a^Chi-square value. ^b^Kruskal Wallis *H* value. ^c^Mann Witney *U* value.

DAD = Disability Assessment for Dementia.

**Supplementary Table 3.** Psychiatric and medical history between patients with and without any hallucinations

|  | **Patients with hallucination**  **(*n* = 23)** | **Patients without hallucination**  **(*n* = 23)** | **Chi-square value** | ***p*** |
| --- | --- | --- | --- | --- |
| Illicit substance use (Y/N) | 3/19 | 4/19 | .121 | .728 |
| Heavy drinking (Y/N) | 7/15 | 4/19 | 1.267 | .260 |
| Smoking (Y/N) | 9/13 | 7/16 | .538 | .463 |
| Psychiatrist consultation (Y/N) | 13/9 | 12/11 | .218 | .641 |
| Antidepressant (Y/N) | 7/16 | 4/19 | 1.075 | .300 |
| Antipsychotic (Y/N) | 7/16 | 3/20 | 2.044 | .153 |
| Blood Pressure medication (Y/N) | 3/20 | 4/19 | .168 | .681 |
| Cholinesterase Inhibition (Y/N) | 2/21 | 1/22 | .357 | .550 |
| Antiepileptic (Y/N) | 1/22 | 1/22 | .000 | 1.00 |
| Parkinson’s medication (Y/N) | 2/21 | 0/23 | 2.091 | .148 |
| ALS medication (Y/N) | 1/22 | 0/23 | 1.022 | .312 |

**Supplementary Table 4.** Psychiatric and medical history between *C9orf72* repeat expansion carriers with and without hallucination

|  | ***C9*+ Patients with hallucination**  **(*n* = 10)** | ***C9*+ Patients without hallucination**  **(*n* = 9)** | **Chi-square value** | ***p*** |
| --- | --- | --- | --- | --- |
| Illicit substance use (Y/N) | 1/9 | 0/9 | .950 | .330 |
| Heavy drinking (Y/N) | 3/7 | 0/9 | 3.206 | .073 |
| Smoking (Y/N) | 3/7 | 2/7 | .148 | .701 |
| Psychiatrist consultation (Y/N) | 7/3 | 2/7 | 4.337 | .037 |
| Antidepressant (Y/N) | 2/8 | 0/9 | 2.012 | .156 |
| Antipsychotic (Y/N) | 5/5 | 0/9 | 6.107 | .013 |
| Blood Pressure medication (Y/N) | 2/8 | 3/6 | .434 | .510 |
| Cholinesterase Inhibition (Y/N) | 0/10 | 0/9 | - | - |
| Antiepileptic (Y/N) | 0/10 | 0/9 | - | - |
| Parkinson’s medication (Y/N) | 0/10 | 0/9 | - | - |
| ALS medication (Y/N) | 1/9 | 0/9 | .950 | .330 |

**Supplementary Table 5.** Psychiatric and medical history between patients with and without visual hallucination

|  | **Patients with visual hallucination**  **(*n* = 8)** | **Patients without any hallucination**  **(*n* = 8)** | **Chi-square value** | ***p*** |
| --- | --- | --- | --- | --- |
| Illicit substance use (Y/N) | 1/7 | 2/6 | .410 | .522 |
| Heavy drinking (Y/N) | 2/6 | 2/6 | .00 | 1.00 |
| Smoking (Y/N) | 4/4 | 4/4 | .00 | 1.00 |
| Psychiatrist consultation (Y/N) | 5/3 | 4/4 | .254 | .614 |
| Antidepressant (Y/N) | 2/6 | 0/8 | 2.286 | .131 |
| Antipsychotic (Y/N) | 0/8 | 0/8 | - | - |
| Blood Pressure medication (Y/N) | 1/7 | 1/7 | .00 | 1.00 |
| Cholinesterase Inhibition (Y/N) | 1/7 | 0/8 | 1.067 | .302 |
| Antiepileptic (Y/N) | 0/8 | 0/8 | - | - |
| Parkinson’s medication (Y/N) | 1/7 | 0/8 | 1.067 | .302 |
| ALS medication (Y/N) | 0/8 | 0/8 | - | - |

**Supplementary Table 6.** Neuropsychological test performance between *C9orf72* repeat expansion carriers with and without hallucination

| **Function** | ***C9*+ Patients with hallucination**  **(*n* = 10)** | ***C9*+ Patients without hallucination**  **(*n* = 9)** | **Healthy Controls**  **(*n* = 10)** | ***H*** | ***p*** | **Post-hoc** | ***p*** |
| --- | --- | --- | --- | --- | --- | --- | --- |
| **ACE** |  |  |  |  |  |  |  |
| Total | 64.96(17.17) | 81.77(9.82) | 96.50(2.99) | 21.599 | <.001 | CRL>HAL, NONHAL | <.001 |
| Attention | 14.90(2.22) | 16.84(0.82) | 17.40(1.26) | 10.432 | .005 | - | - |
| Memory | 14.60(7.00) | 20.89(4.23) | 25.40(1.07) | 16.857 | <.001 | CRL>HAL | <.001 |
| Fluency | 5.00(3.02) | 7.67(3.35) | 13.00(1.15) | 20.805 | <.001 | CRL>HAL, NONHAL | <.001 |
| Language | 18.28(4.93) | 21.68(3.22) | 25.20(0.92) | 16.483 | <.001 | CRL>HAL | <.001 |
| Visuospatial | 12.53(2.97) | 14.79(1.72) | 15.50(0.97) | 8.625 | .013 | - | - |
| **Attention** |  |  |  |  |  |  |  |
| TMT-A (seconds) | 80.20(56.33) | 43.78(22.73) | 30.60(5.19) | 14.512 | <.001 | CRL>HAL | <.001 |
| Forward Digit Span (Max) | 5.50(1.35) | 6.13(1.55) | 6.80(1.48) | 3.294 | .193 | - | - |
| **Working Memory** |  |  |  |  |  |  |  |
| Backward Digit Span (Max) | 3.00(1.25) | 3.88(1.36) | 4.70(0.95) | 8.265 | .016 | - | - |
| **Language** |  |  |  |  |  |  |  |
| SYDBAT- Naming | 18.11(4.81) | 21.38(5.78) | 27.22(2.11) | 12.845 | .002 | - | - |
| SYDBAT- Repetition | 25.33(7.45) | 25.00(6.52) | 29.89(0.33) | 6.159 | .046 | - | - |
| SYDBAT- Comprehension | 24.00(2.87) | 26.88(3.52) | 28.78(2.05) | 10.771 | .005 | - | - |
| SYDBAT- Semantics | 21.33(4.18) | 26.00(2.33) | 27.44(1.88) | 12.817 | .002 | - | - |
| **Visuospatial Function** |  |  |  |  |  |  |  |
| RCF Copy (Raw) | 22.69(10.77) | 24.56(8.42) | 33.00(3.23) | 8.646 | .013 | - | - |
| **Executive Functions** |  |  |  |  |  |  |  |
| Hayling Total (SS) | 2.60(1.52) | 4.13(2.23) | 6.20(0.79) | 11.514 | .003 | - | - |
| TMT-B (errors) | 1.80(1.92) | 0.63(1.19) | 0.20(0.63) | 6.467 | .039 | - | - |
| TMT-B-A (seconds) | 207.40(101.17) | 79.00(45.92) | 43.50(19.23) | 17.744 | <.001 | CRL>HAL | <.001 |
| **Visual Memory** |  |  |  |  |  |  |  |
| RCF 3-minute delay | 7.57(5.11) | 11.31(9.01) | 16.70(4.81) | 7.644 | .022 | - | - |
| **Verbal Memory** |  |  |  |  |  |  |  |
| RAVLT 30-minute delay | 5.50(3.56) | 3.17(2.71) | 10.50(2.80) | 12.411 | .002 | - | - |
| **Emotion Processing** |  |  |  |  |  |  |  |
| FAST |  |  |  |  |  |  |  |
| Negative Emotions | 13.83(5.31) | 16.63(4.66) | 20.60(3.75) | 7.749 | .021 | - | - |
| Positive Emotions | 9.00(2.00) | 10.75(1.58) | 11.30(1.06) | 5.687 | .058 | - | - |
| FADT | 33.00(3.95) | 36.00(3.38) | 35.90(1.10) | 3.754 | .153 | - | - |
|  |  |  |  |  |  |  |  |
| FIDT | 29.67(4.80) | 33.25(6.76) | 35.00(4.30) | 3.347 | .188 | - | - |

Significance set at *p* < .001.

Means (Standard Deviation). H = Kruskal Wallis’ *H* value.

ACE = Addenbrooke’s Cognitive Examination; CRL = Healthy controls; FADT = Face Affect Discrimination Task; FAST = Face Affect Selection Task; FIDT = Face Identity Discrimination Task; HAL = Hallucination group; Hayling = Hayling Sentence Completion Test; NONHAL = Non-hallucination group; RAVLT = Rey Auditory Verbal Learning Test; RCF = Rey Complex Figure; SS = Scaled Score; SYDBAT = Sydney Language Battery; TMT = Trail Making Test.

**Supplementary Table 7.** Neuropsychological test performance between patients with and without visual hallucination

| **Function** | **Healthy Controls**  **(*n* = 8)** | **Patients with visual hallucination**  **(*n* = 8)** | **Patients without any hallucination**  **(*n* = 8)** | ***H*** | ***p*** | **Post-hoc** | ***p*** |
| --- | --- | --- | --- | --- | --- | --- | --- |
| **ACE** |  |  |  |  |  |  |  |
| Total | 96.75(1.98) | 73.80(8.55) | 69.28(12.45) | 15.598 | <.001 | CRL>HAL, NONHAL | <.001 |
| Attention | 17.75(0.46) | 13.27(2.87) | 15.56(1.41) | 14.703 | <.001 | CRL>HAL | <.001 |
| Memory | 25.13(1.46) | 18.50(3.96) | 18.00(3.25) | 14.863 | <.001 | CRL>HAL, NONHAL | <.001 |
| Fluency | 12.63(1.19) | 6.38(3.74) | 4.38(3.50) | 15.232 | <.001 | CRL>HAL, NONHAL | <.001 |
| Language | 25.50(0.53) | 22.00(2.61) | 18.47(6.42) | 14.108 | <.001 | CRL>HAL, NONHAL | <.001 |
| Visuospatial | 15.75(0.46) | 13.76(1.03) | 13.14(2.63) | 9.014 | .011 | - | - |
| **Attention** |  |  |  |  |  |  |  |
| TMT-A (seconds) | 26.80(5.54) | 49.50(20.41) | 62.43(26.21) | 8.550 | .014 | - | - |
| Forward Digit Span (Max) | 8.20(0.45) | 5.88(0.99) | 6.71(1.70) | 8.348 | .015 | - | - |
| **Working Memory** |  |  |  |  |  |  |  |
| Backward Digit Span (Max) | 5.20(0.45) | 3.38(0.52) | 4.43(2.37) | 7.394 | .025 | - | - |
| **Language** |  |  |  |  |  |  |  |
| SYDBAT- Naming | 27.60(2.07) | 22.13(2.85) | 20.14(7.40) | 7.389 | .025 | - | - |
| SYDBAT- Repetition | 30.00(0.00) | 28.71(1.50) | 25.57(6.60) | 4.342 | .114 | - | - |
| SYDBAT- Comprehension | 28.60(2.61) | 26.38(2.83) | 24.88(4.26) | 5.023 | .081 | - | - |
| SYDBAT- Semantics | 27.80(2.77) | 23.75(3.41) | 25.29(3.04) | 5.230 | .073 | - | - |
| **Visuospatial Function** |  |  |  |  |  |  |  |
| RCF Copy (Raw) | 31.40(3.58) | 24.00(5.77) | 23.94(9.04) | 4.817 | .090 | - | - |
| **Executive Functions** |  |  |  |  |  |  |  |
| Hayling Total (SS) | 6.20(0.45) | 3.00(1.41) | 2.33(2.16) | 9.463 | .009 | - | - |
|  |  |  |  |  |  |  |  |
| TMT-B (errors) | 0.00(0.00) | 3.17(1.94) | 0.60(1.34) | 10.457 | .005 | - | - |
|  |  |  |  |  |  |  |  |
| TMT-B-A (seconds) | 30.40(16.76) | 169.00(92.34) | 147.14(110.21) | 9.590 | .008 | - | - |
| **Visual Memory** |  |  |  |  |  |  |  |
| RCF 3-minute delay | 16.20(3.88) | 6.83(3.92) | 6.13(3.38) | 9.966 | .007 | - | - |
| **Verbal Memory** |  |  |  |  |  |  |  |
| **RAVLT 30-minute delay** | 11.40(2.70) | 3.50(4.46) | 5.25(0.96) | 8.535 | .014 | - | - |
|  |  |  |  |  |  |  |  |
| **Emotion Processing** |  |  |  |  |  |  |  |
| FAST |  |  |  |  |  |  |  |
| Negative Emotions | 19.00(5.10) | 14.63(4.47) | 15.17(3.06) | 2.447 | .294 | - | - |
| Positive Emotions | 12.00(0.00) | 8.75(3.20) | 10.33(1.51) | 5.639 | .060 | - | - |
| FADT | 36.25(1.89) | 28.50(6.61) | 31.67(5.92) | 6.675 | .036 | - | - |
| FIDT | 35.50(4.20) | 28.38(5.66) | 28.83(4.02) | 4.937 | .085 | - | - |

Significance set at *p* < .001.

Means (Standard Deviation). H = Kruskal Wallis’ *H* value.

ACE = Addenbrooke’s Cognitive Examination; CRL = Healthy controls; FADT = Face Affect Discrimination Task; FAST = Face Affect Selection Task; FIDT = Face Identity Discrimination Task; HAL = Hallucination group; Hayling = Hayling Sentence Completion Test; NONHAL = Non-hallucination group; RAVLT = Rey Auditory Verbal Learning Test; RCF = Rey Complex Figure; SS = Scaled Score; SYDBAT = Sydney Language Battery; TMT = Trail Making Test.

**Supplementary Table 8.** Behavioural and neuropsychiatric variables between patients with and without any hallucinations

|  | **Patients with hallucination**  **(*n* = 23)** | **Patients without hallucination**  **(*n* = 23)** | ***t*** | ***p*** |
| --- | --- | --- | --- | --- |
| **NPI** |  |  |  |  |
| Agitation | 3.18(3.53) | 1.78(2.58) | 1.525 | .135 |
| Elation | 1.14(3.06) | 0.65(1.70) | .661 | .512 |
| Disinhibition | 4.09(5.12) | 2.48(2.47) | 1.337^a^ | .191 |
| Sexual Interest | 0.00(.00) | 0.00(.00) | - | - |
| Apathy | 5.18(4.06) | 4.96(4.17) | .184 | .855 |
| Irritability | 2.27(3.21) | 1.43(2.19) | 1.018^a^ | .315 |
| Abnormal Motor Behaviour | 3.73(4.34) | 2.09(3.18) | 1.441^a^ | .158 |
| Sleep | 1.82(3.63) | 1.48(2.13) | .385 | .702 |
| Appetite | 2.45(1.63) | 1.78(1.65) | 1.376 | .176 |
| **DASS** |  |  |  |  |
| Depression | 14.13(11.51) | 9.71(8.33) | 1.354 | .184 |
| Anxiety | 9.63(10.26) | 6.10(6.77) | 1.260 | .216 |
| Stress | 13.00(12.80) | 11.33(9.24) | .461 | .648 |

Means (standard deviation). ^a^Equal variance not assumed.

DASS = Depression Anxiety Stress Scale; NPI = Neuropsychiatric Inventory.

**Supplementary Table 9.** Behavioural and neuropsychiatric variables between *C9orf72* repeat expansion carriers with and without hallucination

|  | ***C9*+ Patients with hallucination**  **(*n* = 10)** | ***C9*+ Patients without hallucination**  **(*n* = 9)** | ***U*** | ***p*** |
| --- | --- | --- | --- | --- |
| **NPI** |  |  |  |  |
| Agitation | 2.10(2.42) | .67(2.00) | 25.00 | .113 |
| Elation | .80(2.53) | .89(2.67) | 44.50 | .968 |
| Disinhibition | 4.90(6.12) | 2.78(1.86) | 40.00 | .720 |
| Sexual Interest | 0(.00) | 0(.00) | 45.00 | 1.000 |
| Apathy | 3.80(3.22) | 4.11(3.33) | 44.00 | .968 |
| Irritability | 2.10(3.60) | 1.78(2.17) | 39.500 | .661 |
| Abnormal Motor Behaviour | 3.60(4.22) | 2.78(4.35) | 38.500 | .604 |
| Sleep | 0.60(1.90) | 1.33(2.65) | 39.500 | .661 |
| Appetite | 2.20(1.55) | 2.00(1.73) | 42.000 | .842 |
| **DASS** |  |  |  |  |
| Depression | 12.25(14.12) | 11.00(9.97) | 29.500 | .798 |
| Anxiety | 10.25(12.49) | 10.00(8.68) | 29.000 | .798 |
| Stress | 12.00(14.30) | 15.00(11.76) | 27.000 | .645 |

Means (standard deviation). *U* = Mann-Whitney *U* Value.

DASS = Depression Anxiety Stress Scale; NPI = Neuropsychiatric Inventory.

**Supplementary Table 10.** Behavioural and neuropsychiatric variables between patients with and without visual hallucination

|  | **Patients with visual hallucination**  **(*n* = 8)** | **Patients without any hallucination**  **(*n* = 8)** | ***U*** | ***p*** |
| --- | --- | --- | --- | --- |
| **NPI** |  |  |  |  |
| Agitation | 2.13(2.75) | 0.38(.74) | 21.50 | .279 |
| Elation | 1.63(4.21) | 1.13(2.80) | 31.50 | .959 |
| Disinhibition | 2.88(4.79) | 1.75(2.05) | 29.00 | .798 |
| Sexual Interest | 0(.00) | 0(.00) | 32.00 | 1.000 |
| Apathy | 4.00(3.59) | 5.25(4.68) | 28.00 | .721 |
| Irritability | 1.50(2.78) | 0.50(.76) | 31.00 | .959 |
| Abnormal Motor Behaviour | 1.75(2.92) | 0.50(1.41) | 24.00 | .442 |
| Sleep | 0.88(1.46) | 0.63(1.41) | 28.00 | .721 |
| Appetite | 2.38(1.77) | 1.50(1.60) | 20.00 | .234 |
| **DASS** |  |  |  |  |
| Depression | 13.33(7.87) | 12.75(9.32) | 23.00 | .950 |
| Anxiety | 7.67(7.74) | 9.75(8.71) | 20.00 | .662 |
| Stress | 11.00(10.94) | 14.00(11.90) | 22.500 | .852 |

Means (standard deviation). *U* = Mann-Whitney *U* Value.

DASS = Depression Anxiety Stress Scale; NPI = Neuropsychiatric Inventory.


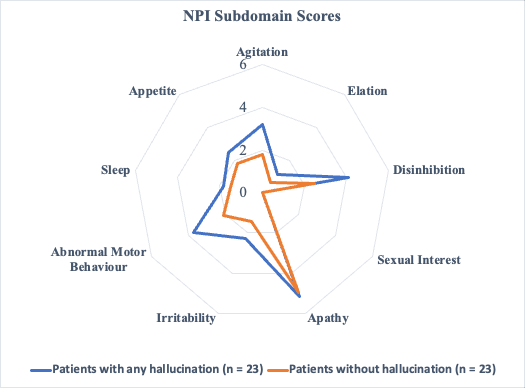


**Supplementary** **Figure 1.** A radar plot demonstrating the mean Neuropsychiatric Inventory (NPI) subdomain scores between patients with and without any hallucinations


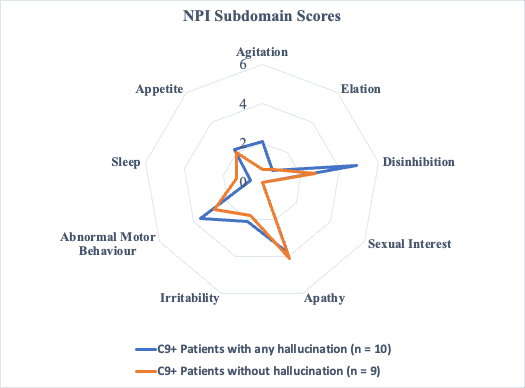


**Supplementary** **Figure 2.** A radar plot demonstrating the mean Neuropsychiatric Inventory (NPI) subdomain scores between *C9orf72* repeat expansion carriers with and without hallucination


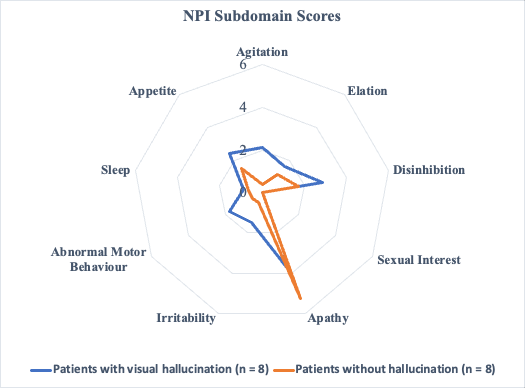


**Supplementary** **Figure 3.** A radar plot demonstrating the mean Neuropsychiatric Inventory (NPI) subdomain scores between patients with and without visual hallucinations

**
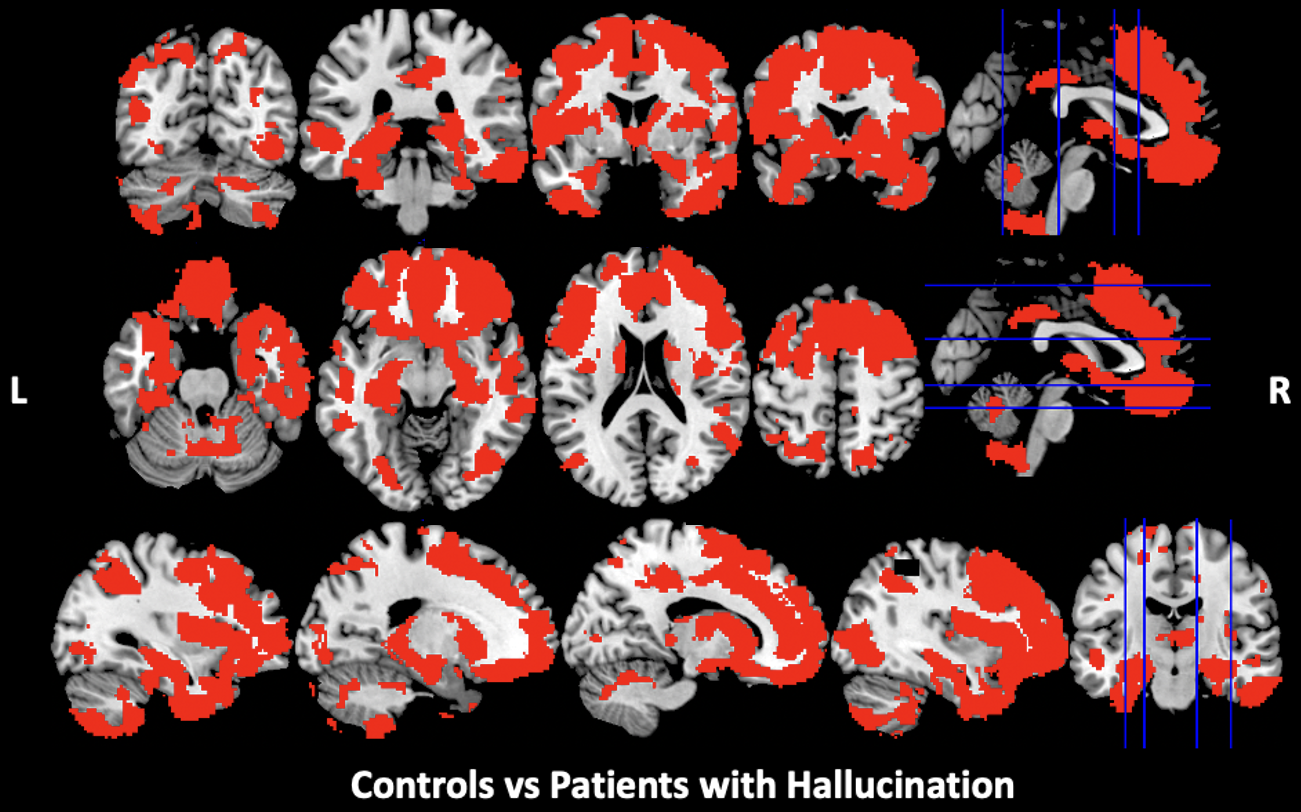
**

**Supplementary** **Figure 4.** Voxel-based morphometry analyses showing whole brain atrophy in contrasts between patients with any type of hallucinations (*n* = 23) and controls (*n* = 23). Shaded voxels show regions that were significant in the analyses after correction for False Discovery Rate (FDR) at *p* < 0.05 with a cluster threshold of 100 contiguous voxels. L = Left; R = Right.

**
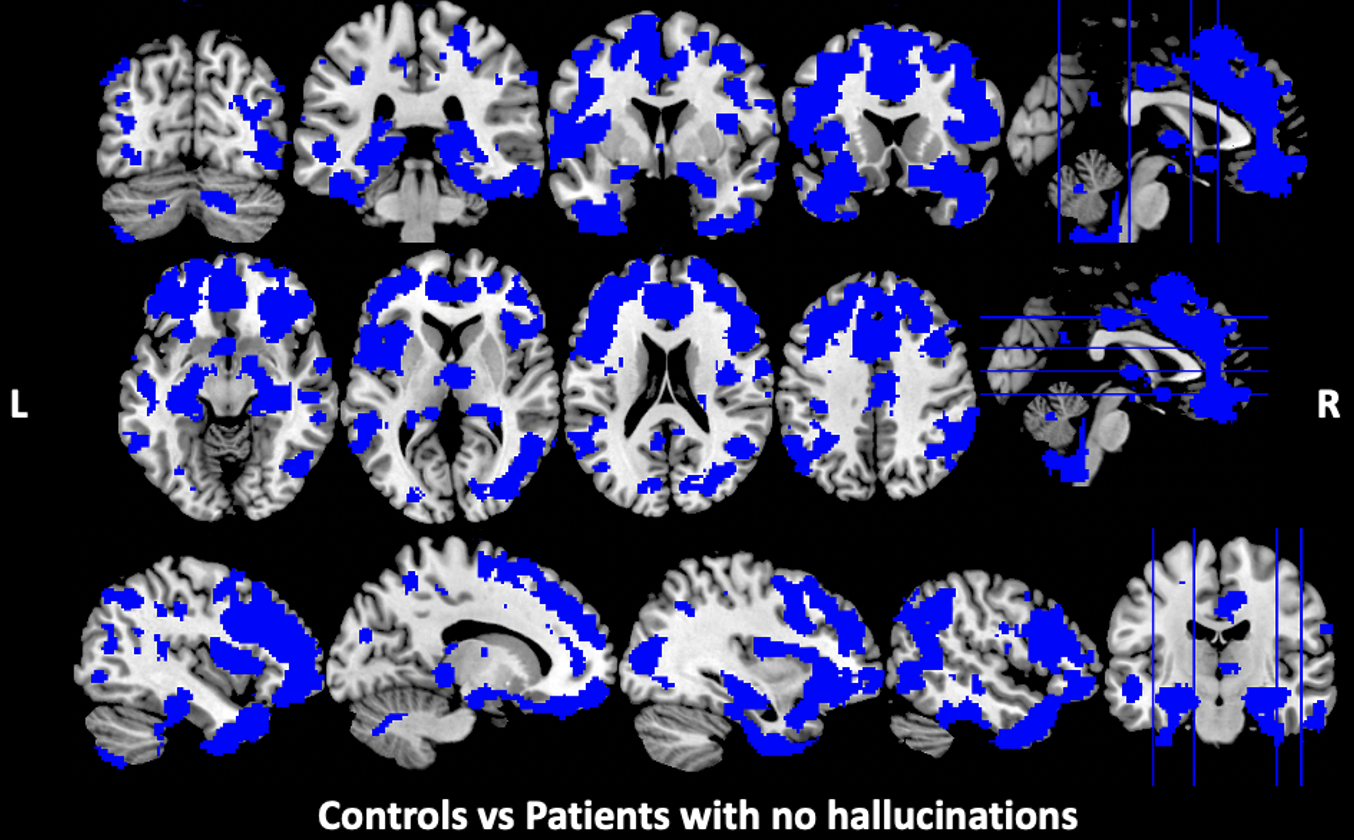
**

**Supplementary** **Figure 5.** Voxel-based morphometry analyses showing whole brain atrophy in contrasts between patients without any hallucinations (*n* = 23) and controls (*n* = 23). Shaded voxels show regions that were significant in the analyses after correction for False Discovery Rate (FDR) at *p* < 0.05 with a cluster threshold of 100 contiguous voxels. L = Left; R = Right.

**
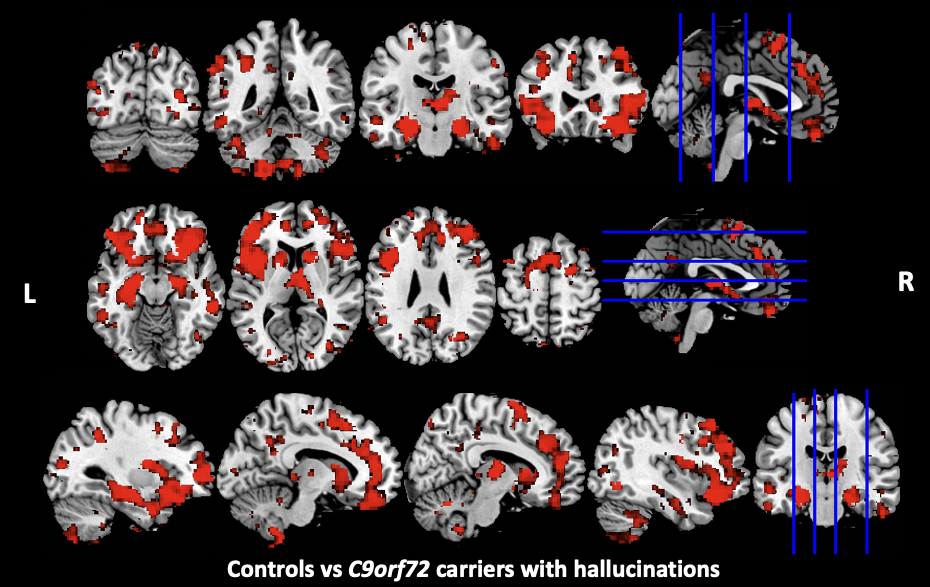
**

**Supplementary** **Figure 6.** Voxel-based morphometry analyses showing whole brain atrophy in contrasts between *C9orf72* carriers with any type of hallucinations (*n* = 10) and controls (*n* = 10). Shaded voxels show regions that were significant in the analyses at the threshold of *p* < 0.005 (uncorrected) with a cluster threshold of 100 contiguous voxels. L = Left; R = Right.

**
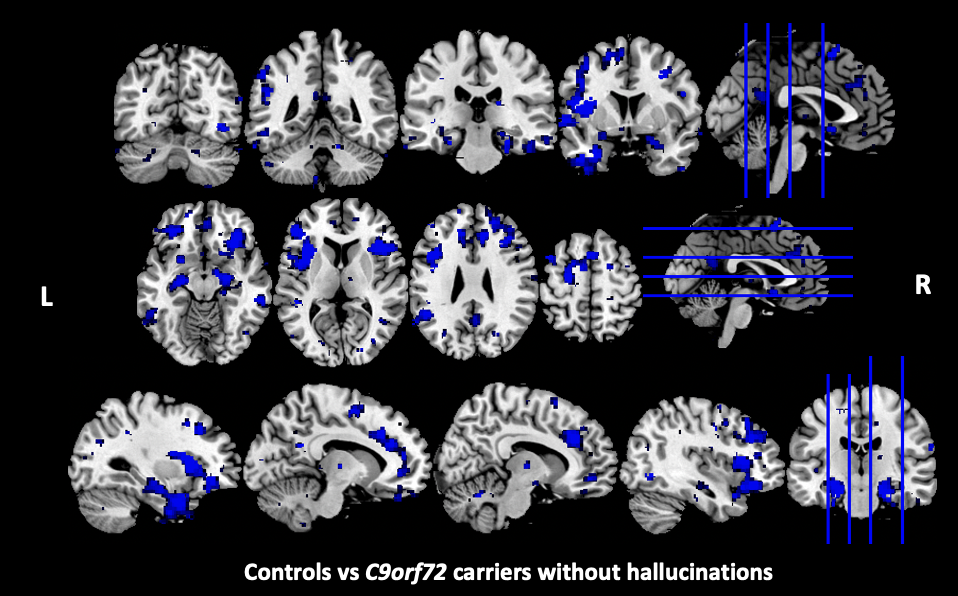
**

**Supplementary** **Figure 7.** Voxel-based morphometry analyses showing whole brain atrophy in contrasts between *C9orf72* carriers without hallucinations (*n* = 9) and controls (*n* = 10). Shaded voxels show regions that were significant in the analyses at the threshold of *p* < 0.005 (uncorrected) with a cluster threshold of 100 contiguous voxels. L = Left; R = Right.


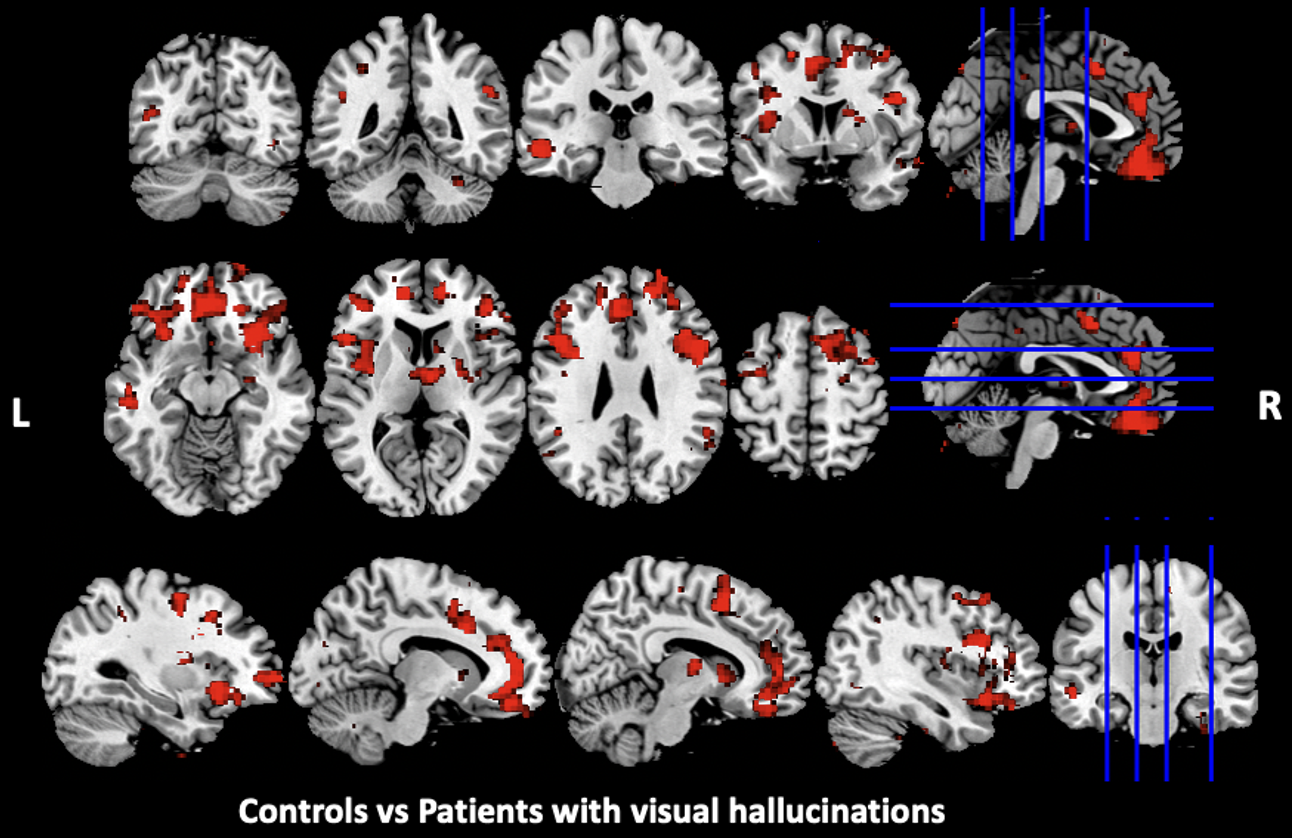


**Supplementary** **Figure 8.** Voxel-based morphometry analyses showing whole brain atrophy in contrasts between patients with visual hallucinations (*n* = 8) and controls (*n* = 8). Shaded voxels show regions that were significant in the analyses at the threshold of *p* < 0.005 (uncorrected) with a cluster threshold of 100 contiguous voxels. L = Left; R = Right.

**
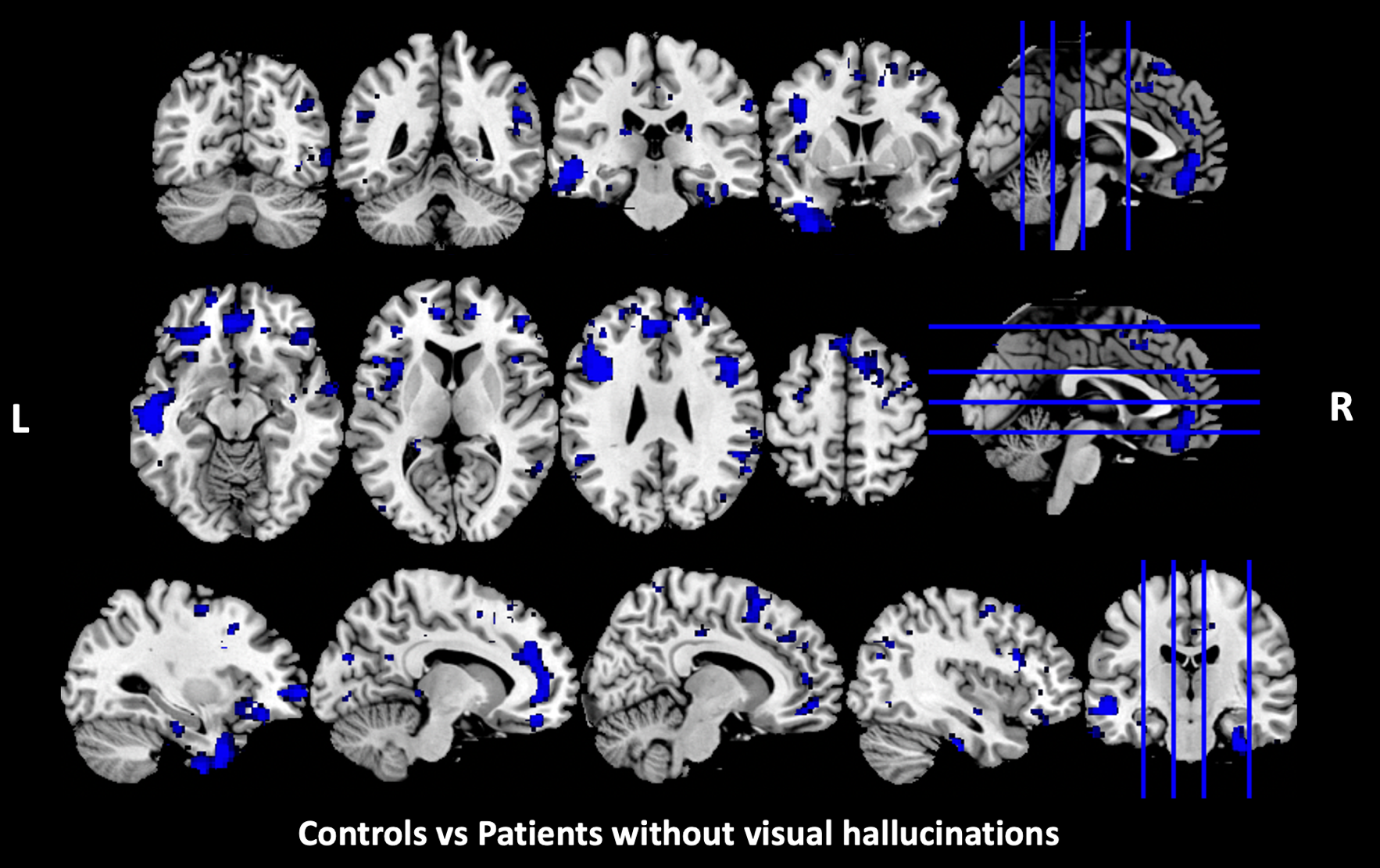
**

**Supplementary** **Figure 9.** Voxel-based morphometry analyses showing whole brain atrophy in contrasts between patients without visual hallucinations (*n* = 8) and controls (*n* = 8). Shaded voxels show regions that were significant in the analyses at the threshold of *p* < 0.005 (uncorrected) with a cluster threshold of 100 contiguous voxels. L = Left; R = Right.
